# P003. NSAIDs for symptomatic treatment of headache

**DOI:** 10.1186/1129-2377-16-S1-A162

**Published:** 2015-09-28

**Authors:** Giannapia Affaitati, Paolo Martelletti, Mariangela Lopopolo, Claudio Tana, Francesca Massimini, Francesco Cipollone, Domenico Lapenna, Maria Adele Giamberardino, Raffaele Costantini

**Affiliations:** Headache Centre, Department of Medicine and Science of Aging, “G. D'Annunzio” University of Chieti, Chieti, Italy; Department of Clinical and Molecular Medicine, Regional Referral Headache Centre, “Sant'Andrea” Hospital, “Sapienza” University, Rome, Italy; Internal Medicine Unit, Guastalla Hospital, AUSL Reggio Emilia, Reggio Emilia, Italy; Institute of Clinical Pathology, “G. D'Annunzio” University of Chieti, Chieti, Italy; Institute of Surgical Pathology, “G. D'Annunzio” University of Chieti, Chieti, Italy

## Background and aims

Clinical observations suggest that the use of non-steroidal anti-inflammatory drugs (NSAIDs) for symptomatic treatment of headache is not in line with recommendations by international guidelines [1]. The aim of the study was to evaluate NSAIDs use for episodic headache at the Headache Centre of the Chieti University in the period: January 2000-February 2013.

## Methods

A retrospective evaluation was carried out on 6,443 records of episodic migraine (n=2,330), tension-type headache (TTH) (n=807) and migraine + TTH (n=3,306) sufferers relative to NSAID use for the acute attack: type of NSAID/s; uni- or poly-therapy (one or more NSAID/s in different attacks) and NSAID efficacy (subjective scale: complete (C), partial (P) or absent (A) pain relief at 2 hours), at the first visit and 1-year follow-up.

## Results

In migraine patients, 80% had been NSAID users in the past year. The three most frequently employed molecules were: nimesulide (57%), ketoprofen (25%) and ibuprofen (24%). Complete vs. incomplete/absent efficacy was significantly higher for all three (p < 0.0001). NSAIDs were replaced with triptans in 53% of the patients at the first visit; after 1 year: a significant spontaneous return to NSAIDs occurred in 56% of the cases (p < 0.0005) for inadequate efficacy/side effects (62%) or difficulty in obtaining prescription (38%) of triptans from the family doctor. In TTH patients, 90% were NSAID users; preferences were: nimesulide (48%), ketoprofen (47%) and diclofenac (19%); complete vs. incomplete/absent efficacy was significantly higher for the first two (p < 0.02). Replacement with analgesics was performed in 24% of the patients at the first visit; at one-year follow-up a spontaneous return to NSAIDs occurred in 29% of the cases for inadequate efficacy of the non-NSAID therapy. In Migraine + TTH patients who were not able to distinguish the nature of their attack at the beginning of the pain, 89% were NSAID users; the three most frequently employed molecules were: nimesulide (44%), ibuprofen (42%) and ketoprofen (38%); complete vs. incomplete/absent efficacy was significantly higher for all three (0.001 < p < 0.0001). Replacement with analgesics was prescribed to 31% of the patients at the first visit; at one-year follow-up a spontaneous return to NSAIDs occurred in 37% of them for inadequate efficacy of the non-NSAID therapy.

## Conclusions

NSAID use in episodic headache is higher than could be hypothesized based on guidelines [2]. NSAID role/efficacy, particularly in migraine, should be better understood. A higher degree of sensitization towards different treatment options for headache should also be promoted in the medical environment [3].

Written informed consent to publish was obtained from the patient(s).
